# Lemon Juice in Acute Rheumatism

**Published:** 1852-09

**Authors:** T. D. Lee

**Affiliations:** New York


*Lemon Juice in Acute Rheumatism.* By T. D. Lee, M. D., of New York.—*Case* 1.—William Coleman, fifty-one years of age, has had frequent and severe attacks of acute rheumatism for the last fourteen years.

April 30th, 1852.—Patient has not been able to remove from his bed for the last four weeks; and he is unable to sleep by night on account of acute pain; his hands, knees, and feet are very much swollen; and he is constantly growing worse. Patient has heretofore had attacks which have lasted three or four months. At 8 o'clock, P. M., patient commenced taking lemon juice, fresh from lemons; a tablespoonful in twice the quantity of cold water with a little sugar, every hour.

May 1st, 9 o'clock, A. M.,—Patient sitting up endeavoring to shave himself. Says he feels better in every respect, and has slept quietly for four hours, which he has not done before for four weeks. Has taken six ounces of the juice. Same regimen to be continued as before.

May 2d, 9 o'clock, A. M.—Patient has slept well during the night, and is sitting up eating his breakfast. Six ounces taken. Says "lemon juice goes right to the spot."

May 3d, A. M.—Free from pain, and only complains of soreness in his feet. Same quantity as before.

May 4th.—Still improving, having taken six ounces regularly.

May 5th.—Comfortable, and taking the same quantity of lemon.

May 6th.—Doing well, still taking the same quantity of lemon, having no disposition to leave it off.

May 7th.—Patient is able to walk about his room, and does not speak of any pain. Lemon continued.

May 8th.—Patient complains only of weakness in walking. Lemon juice discontinued, not having disagreed with the stomach or bowels in the slightest degree.

May 9th.—Patient sleeps well by night; has a good appetite, and is fast gaining strength.

May 10th.—Patient is only waiting for fair weather to go out.

*Case* 2.—Mrs. P., seventeen years of age, has had frequent attacks of acute rheumatism for the last seven years. She is affected with enlargement of the heart. Patient is confined to her bed, and is unable to move or sleep on account of the severe pain she suffers; and wishes to have something prescribed to make her sleep. Patient was advised to try lemon juice for 24 hours, before using any other remedy; to this she assented. Remedy commenced at 10 A. M., as in case 1st.

May 2d.—Patient has slept well, and has much less pain and soreness.

May 3d, 4th and 5th.—Improving. Continue lemon as before.

May 6th.—Free from all appearance of the disease. Lemon discontinued.

May 7th. In this case, as in Case 1st, there appears to be a flexibility of the joints, unusual in recoveries after other modes of treatment.

In a third case similar to Case 1st, the lemon appeared to be equally beneficial. In two other cases, one subacute, the other chronic in type, the lemon juice had an equally good effect.—*N. Y. Med. Jour.*

